# Lotus Sprout Extract Induces Selective Melanosomal Autophagy and Reduces Pigmentation

**DOI:** 10.1111/jocd.16587

**Published:** 2024-09-21

**Authors:** Mikhail Geyfman, Robin Chung, Raymond Boissy, Neil Poloso, Kuniko Kadoya, Prithwiraj Maitra, Rahul Mehta

**Affiliations:** ^1^ Allergan Aesthetics, An AbbVie Company Irvine California USA; ^2^ University of Cincinnati Cincinnati Ohio USA

**Keywords:** autophagy, hyperpigmentation, melanin, melanogenesis, melanosomes

## Abstract

**Background:**

Hyperpigmentation disorders are caused by the excess production and irregular accumulation of melanin. Existing treatments often have limited efficacy and adverse effects, necessitating the development of new skin‐brightening agents. Lotus sprout extract (LSE) was identified as a potential pigment‐correcting agent. However, the active compounds responsible for driving mechanisms related to this activity remain unknown.

**Aims:**

This study aimed to investigate the effects of LSE and its active components, neferine and liensinine, on melanin accumulation and to understand how LSE reduces skin pigmentation.

**Methods:**

Melanin accumulation was analyzed in MNT‐1 human melanoma cells and MelanoDerm human skin equivalents following neferine, liensinine, or LSE treatment. The effects of the compounds on different pathways regulating melanin levels were evaluated by gene expression, biochemical assays, and western blotting. Melanosome ultrastructure was monitored using transmission electron microscopy (TEM).

**Results:**

Neferine and liensinine reduced melanin accumulation in MNT‐1 cells without downregulating melanogenesis‐related genes or inhibiting tyrosinase activity. Instead, these compounds increased autophagic flux, suggesting that the reduction in pigmentation was due to increased melanin degradation. LSE also reduced melanin accumulation and activated autophagy in normal human melanocytes and MelanoDerm tissue. Autophagosomes induced by LSE treatment contained only melanosomes, and structural changes in melanosomes suggested that LSE may disrupt melanosome maturation.

**Conclusion:**

This study revealed a novel mechanism for LSE, neferine, and liensinine in reducing pigmentation, potentially through the induction of autophagy and subsequent melanosome degradation. These findings suggest that LSE and its enriched bioactive compounds could be promising agents for treating hyperpigmentation.

## 1 Introduction

Visible skin color depends on the amount, type, and distribution of melanin, which normally protects the skin from ultraviolet radiation‐associated damage. However, excess production and irregular accumulation of melanin in the skin leads to hyperpigmentation, a bothersome aesthetic concern [1]. Commonly used treatment options for hyperpigmentation include topical agents that act on one or more pathways involved in melanin production, distribution, or degradation [2]. The most widely used products to treat hyperpigmentation often have limited efficacy or are associated with adverse effects including hypopigmentation, dermatitis, or achromoderma [3]. These limitations and concerns highlight the need for new skin‐brightening agents [2].

The total melanin content of the skin is regulated by several distinct processes that serve as potential points of intervention for the treatment of hyperpigmentation. Melanin is synthesized and stored within melanosomes, specialized membrane‐bound organelles produced by melanocytes at the dermal–epidermal junction. Melanosome biogenesis, known as melanogenesis, is regulated by numerous signaling pathways and involves the coordinated expression and delivery of specific structural proteins, enzymes, and substrates to facilitate the formation of fully pigmented mature melanosomes [4]. For instance, the activity of tyrosinase (TYR), tyrosinase‐related protein‐1 (TYRP1), and dopachrome tautomerase (DCT) is critical to pigment synthesis, while the proteins PMEL and MLANA promote melanosome maturation [5]. Melanosome maturation occurs in four stages: stages I and II melanosomes establish their structural foundations, while stages III and IV melanosomes develop melanin and are enriched with pigmentation [6]. Mature melanosomes are transported through dendritic projections of melanocytes and into connecting keratinocytes. Melanin can then either be indirectly shed from the skin through desquamation or directly degraded within cells. In contrast to melanin biosynthesis, which is relatively well characterized, much less is known about pathways that control melanin turnover [5]. Recently, autophagy, a catabolic process for degrading and recycling damaged or excess cellular components, is recognized as an important mediator of melanin turnover, the degradation, and cycling of melanin within keratinocytes [5]. Thus, autophagy represents an actionable target for treating hyperpigmentation [7, 8].

Plant extracts are a potent source of bioactive secondary metabolites such as flavonoids, phenols, and alkaloids, many of which have shown benefits for the treatment of dermatologic conditions, including hyperpigmentation [9]. Extracts from plants used in traditional medicine practices are an attractive option to consider for new cosmetic ingredients as they are a source of active compounds known to have cosmetic benefits with a reasonably perceived tolerability [10]. *Nelumbo nucifera*, also known as lotus, is an aquatic plant cultivated and consumed in countries across Asia. While many of the individual parts of the lotus plant are used in traditional Chinese and Ayurvedic medicine to treat several different conditions, the pathways that specific bioactive compounds act on are not widely studied. Recently, extracts from different parts of the lotus plant have been explored as potential treatments for hyperpigmentation [11, 12, 13, 14].

Neferine and liensinine are bisbenzylisoquinoline alkaloids that are highly abundant in lotus sprout extract (LSE) that have several therapeutic properties, such as antioxidant and anti‐inflammatory activity via mitogen‐active protein kinase and nuclear‐factor kappa B pathways [15, 16, 17]. However, their potential effects on hyperpigmentation have not been explored. Given that neferine and liensinine are bioactive compounds concentrated in LSE with therapeutic properties relevant to hyperpigmentation, we hypothesized that they could contribute to the depigmenting effects previously reported for LSE. In this study, we investigated the effects of LSE and its active constituents, neferine and liensinine, on melanin accumulation to understand how LSE reduces skin pigmentation.

## 2 Methods

### 2.1 Materials

Neferine, liensinine, pepstatin A, and E64d were purchased from Selleck Chemicals (Houston, TX, USA). The synthetic melanin used as standards was purchased from Sigma Aldrich (St. Louis, MO, USA). The LSE used in studies was an extract containing water, 1,2 propanediol, and polysorbate, prepared by a proprietary process to enrich concentrations of neferine and liensinine.

### 2.2 Tissue Culture

Human MNT‐1 melanoma cells (CRL‐3450) were obtained from American Type Culture Collection and cultured in minimum essential media supplemented with 20% heat‐inactivated fetal bovine serum, 10% AIM‐V medium, 20 mM HEPES, 100 μg/mL streptomycin with 100 U/mL penicillin, 0.1 mM nonessential amino acids, and 1 mM sodium pyruvate (all media components from ThermoFisher, Waltham, MA, USA). Cells were maintained at 37°C, 5% CO_2_. Human epidermal melanocytes from darkly pigmented donors (HEMn‐DP cells) were cultured in Medium 254 with a human melanocyte growth supplement (acquired from Gibco, ThermoFisher). MelanoDerm human skin equivalents were purchased from MatTek (MEL‐300‐B; MatTek Corp., Ashland, MA, USA), which comprises melanocytes co‐cultured with keratinocytes, both derived from an African American donor. Melanocytes from darker‐pigmented donors were chosen given the higher incidence of hyperpigmentation in darker racial ethnic groups [18]. In addition, these skin types contain higher concentrations of melanin in these skin types to test for changes in pigmentation following treatments [19]. Tissues were cultured at the air‐liquid interface using EPI‐100‐NMM‐113 media (MatTek Corp.) for 15 days with topical application of LSE every other day.

### 2.3 Melanin Extraction and Quantification

For melanin quantification in MNT‐1 cells, the cells were treated with 2 μL of either vehicle or test compounds using a pipette every 3 days for 7 days total. After treatment, cells were lysed in 350 μL of 1 M NaOH. 50 μL cell supernatants and a series of synthetic melanin standards were transferred to each individual well in a black‐walled assay plate. Absorbance values for samples were read at 490 nm with a Spectramax 340PC microplate reader (Molecular Devices, San Jose, CA, USA), and melanin content was calculated based on values from the synthetic melanin standard curve (Table 1). Each condition was performed in duplicate in each experiment.

For melanin quantification in MelanoDerm cultures, test compounds were topically applied to the surface of the tissues every other day for 15 days. The treated tissues and melanin standards were then solubilized in SOLVABLE aqueous‐based solubilizer (0.4 M sodium hydroxide in water, plus three specialized surfactants; PerkinElmer, 6NE9100) overnight. Tissue supernatants or standards were then analyzed similarly to MNT‐1 cells as described above.

### 2.4 Tyrosinase Activity Assay

Tyrosinase catalytic activity was measured using the Cell‐Free Colorimetric Tyrosinase Inhibitor Assay Kit (Abcam, Cambridge, UK) according to the manufacturer's instructions. Briefly, the test compounds or kojic acid positive control were mixed in the reaction wells with the tyrosinase enzyme and incubated at 25°C for 10 min. The substrate solution was then mixed into the reaction wells, and absorbance was measured at 510 nm. Reactions were performed in duplicates. Tyrosinase inhibition was calculated from the reaction rate relative to the control for all conditions.

### 2.5 RNA Isolation and Quantitative PCR

After 48 h of treatment, RNA was purified from MNT‐1 cells using TRIzol, cDNA synthesis was performed with 400 ng total RNA using the SuperScript III Reverse Transcription Kit, and quantitative PCR reactions were performed using the TaqMan gene expression master mix (all from ThermoFisher). The following TaqMan probes were used (all from ThermoFisher): B2M (Ref #4325797), TYR (Hs00165976_m1), TYRP1 (Hs00167051_m1), DCT (Hs01098278_m1), PMEL (Hs00173854_m1), and MLANA (Hs00194133_m1).

### 2.6 Western Blotting

MNT‐1 cells were treated for either 6 or 48 h prior to harvesting for western blotting. After treatment, cells were washed in PBS and lysed in ice‐cold RIPA buffer (ThermoFisher) supplemented with Halt protease inhibitor cocktail (ThermoFisher). The protein concentration of the cleared lysate was measured using the MicroBCA Assay Kit (ThermoFisher) according to the manufacturer's protocol. Thirty micrograms of protein was resolved by SDS‐PAGE using a 4%–12% bis‐tris gel and transferred to a nitrocellulose membrane (Bio‐Rad; Hercules, CA, USA). Membranes were blocked for 1 h in TBS blocking solution (Li‐Cor; Lincoln, NE, USA) for 1 h, followed by overnight incubation in primary antibody. The following primary antibodies were used: mouse anti‐α Tubulin (diluted 1:5000, cat no. ab7291; Abcam), Phospho‐Beclin‐1 Ser90 (diluted 1:500, cat no. 86455; Cell Signaling Technology, Danvers, MA, USA) Phospho‐Beclin‐1 Ser15 Rabbit mAb (diluted 1:500, cat no.84966; Cell Signaling Technology), and LC3B (diluted 1:650 cat no. 2775; Cell Signaling Technology). All primary antibodies were diluted in TBS blocking solution with 0.2% Tween 20 (Li‐Cor). After washing three times with TBS‐Tween 0.1% (ThermoFisher), the membranes were incubated with IR dye‐labeled secondary antibodies (Li‐Cor) diluted 1:20 000 in TBS blocking solution for 1 h at room temperature. The membranes were washed three times with TBS‐Tween 0.1% and scanned using the Odyssey Infrared Imaging System (Li‐Cor).

### 2.7 Fontana‐Masson Staining

After treatment for 15 days, MelanoDerm cultures were collected and fixed overnight with 10% neutral buffered formalin. Samples were processed, embedded in paraffin blocks, and sectioned. Slides were stained with the Fontana‐Masson Stain Kit (American Mastertech Scientific) according to the manufacturer's protocol. Images were captured using the NanoZoomer digital image scanner (Hamamatsu Photonics Corp., Hamamatsu City, Japan). Hematoxylin–eosin staining was performed using standard methods.

### 2.8 Electron Microscopy

MelanoDerm tissues were fixed in half‐strength Karnovsky fixative and placed at 4°C overnight. The fixative was replaced with 0.2 M cacodylate buffer for 24 h, postfixed with 1% osmium tetroxide containing 1.5% potassium ferrocyanide for 30 min, dehydrated with increasing concentrations of ethanol, and then embedded in Spurr's epoxy. Embedded tissue was sectioned and stained with aqueous solutions of 2% uranyl acetate and 0.3% lead citrate for 15 min. Sections were viewed and photographed using a transmission electron microscope (JEM‐1230; JEOL, Akashima, Japan). Autophagosomes were identified as membrane‐bound vesicles containing cellular material that predominately consisted of melanosomes.

### 2.9 Statistical Analysis

Results are expressed as mean ± standard deviation (SD) for the number of replicates as indicated in the figure legends. Statistical significance was calculated by unpaired Student's *t*‐tests using the QuickCalcs statistical calculator (GraphPad, San Diego, CA, USA). A *p*‐value < 0.05 was considered statistically significant.

## 3 Results

**FIGURE 1:**
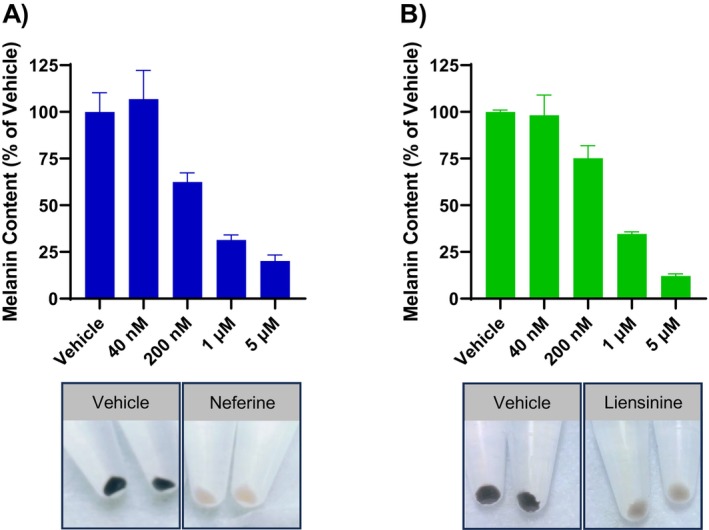
Neferine and liensinine reduce melanin accumulation in MNT‐1 cells. Melanin content after 7 days of treatment with neferine (A) or liensinine (B), relative to vehicle‐treated cells. Images below graph are the pellets from the cells treated with 5 μM neferine (A) or liensinine (B). Data represent mean ± SD, *n* = 2.

We first examined the effects of neferine and liensinine on melanin accumulation in the pigmented human melanoma cell line MNT‐1. Treatment with neferine or liensinine for 7 days produced a dose‐dependent reduction in cellular melanin content in MNT‐1 cells (Figure 1A,B). Accordingly, the pellets from cells treated with 5 μM neferine or liensinine were nearly colorless, in contrast to the visibly dark pigmented pellets from vehicle‐treated cells (Figure 1A,B).

**FIGURE 2:**
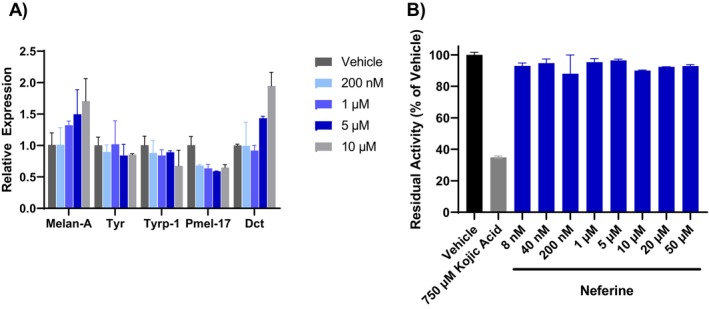
Neferine does not affect melanogenesis gene expression or tyrosinase activity. (A) Normalized gene expression (compared to vehicle) for MNT‐1 cells treated for 48 h with the indicated doses of neferine. Data represent mean ± SD from two technical replicates. (B) Recombinant tyrosinase enzyme activity after treatment with the indicated concentrations of kojic acid (positive control) and neferine. Data represent mean ± SD from two technical replicates.

To understand how these compounds regulate melanin levels, we examined the effect of neferine on the expression of genes related to melanin biogenesis. Neferine did not decrease the expression of *TYR, TYRP1, and DCT* genes involved with melanin synthesis or *PMEL* and *MLANA* genes involved with melanosome formation (Figure 2A). Because the *TYR* gene product, tyrosinase, is a central regulator of melanogenesis and a common target of depigmenting agents, we further evaluated the effect of neferine on tyrosinase catalytic activity. While the well‐established tyrosinase inhibitor kojic acid greatly reduced tyrosinase activity, neferine at concentrations up to 50 μM had no effect (Figure 2B).

**FIGURE 3:**
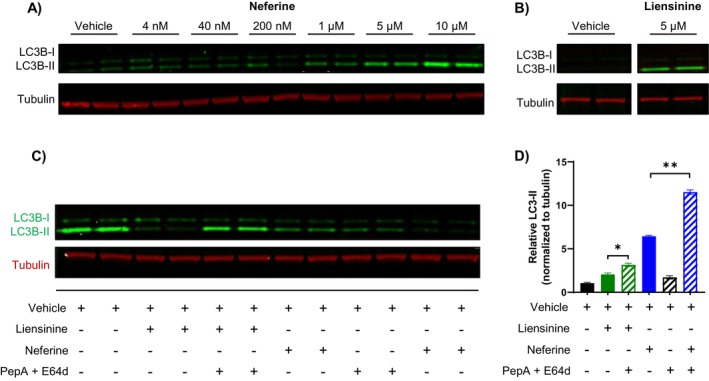
Neferine and liensinine increase autophagic flux. (A, B) Western blot of LC3‐I/II protein in MNT‐1 cells treated with neferine (A) or liensinine (B) for 48 h. Each condition was run in duplicate in neighboring western blot lanes. In (B), lanes unrelated to the experiment were cropped out. (C) Western blot of LC3‐I/II protein in MNT‐1 cells after co‐treatment with neferine or liensinine in the presence or absence of the indicated inhibitors. (D) Quantification of western blot in (C). The LC3‐II intensity was normalized to tubulin and then expressed relative to the vehicle control. Error bars represent mean ± SD of two technical replicates. *p < 0.05, **p < 0.01, by Student's *t*‐test.

Considering that neither pro‐melanogenic gene expression nor tyrosinase activity was inhibited, we explored alternative mechanisms by which pigmentation may be reduced by neferine and liensinine. Given that both compounds have been previously reported to modulate autophagy and growing evidence of autophagy as a regulator of pigmentation, we examined their effects on the autophagosome marker LC3‐II by western blotting [20]. A dose‐dependent induction of LC3‐II levels was observed after 48 h of neferine treatment, reflecting an increase in autophagosome accumulation (Figure 3A). Liensinine at 5 μM also increased LC3‐II levels but to a lesser extent than 5 μM neferine (Figure 3A). To distinguish between an increase in autophagic flux and a blockade in autophagosome degradation, we evaluated the effects of neferine and liensinine on LC3‐II accumulation in the presence or absence of the lysosomal protease inhibitors pepstatin A and E64d. There was a significant increase in LC3‐II accumulation when neferine or liensinine were co‐administered with pepstatin A and E64d, compared to neferine or liensinine alone, implying that autophagic flux was increased (Figure 3B,C).

**FIGURE 4:**
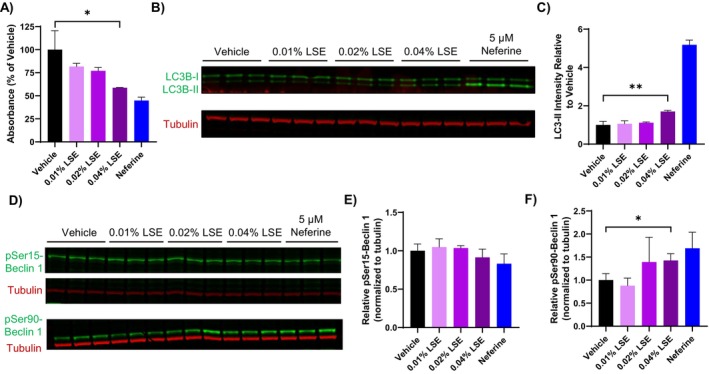
LSE reduces melanin accumulation and induces autophagy in MNT‐1 cells. (A) Melanin content of MNT‐1 cells after treatment as indicated for 7 days. (B) Western blot of LC3‐I/II protein in MNT‐1 cells treated as indicated for 48 h. Each condition was run in triplicate in neighboring western blot lanes. (C) Quantification of western blot in (B). The LC3‐II intensity was normalized to tubulin and then expressed relative to the vehicle control. (D) Western blot of pSer15‐ and pSer90‐Beclin1 protein in MNT‐1 cells treated as indicated for 48 h. (E, F) Quantification of western blots in (D). The target band intensities were normalized to tubulin and then expressed relative to the vehicle control. Error bars represent mean ± SD of three technical replicates. *p < 0.05. **p < 0.01, by Student's *t*‐test.

Because neferine and liensinine are abundant components in LSE, we examined whether LSE treatment reduced pigmentation in MNT‐1 cells by a similar mechanism. A significant decrease in absorbance from melanin was observed at the highest concentration of LSE tested (0.4%) after 5 days of treatment, similar to the effect of neferine (Figure 4A). The reduction in melanin content also corresponded to a significant increase in LC3‐II levels after LSE treatment, although the increase was not as robust as observed with neferine (Figure 4B). To investigate the signaling pathways involved with the induction of autophagy, we monitored phosphorylation of Beclin‐1 at Ser15 and Ser90, phosphorylation sites regulated by mTOR or AMPK, respectively [21, 22]. Treatment with LSE or neferine for 48 h did not affect Ser15 phosphorylation levels, while a significant increase in Ser90 phosphorylation was observed (Figure 4B,C). This suggests that LSE induces autophagy through a mechanism involving AMPK.

**FIGURE 5:**
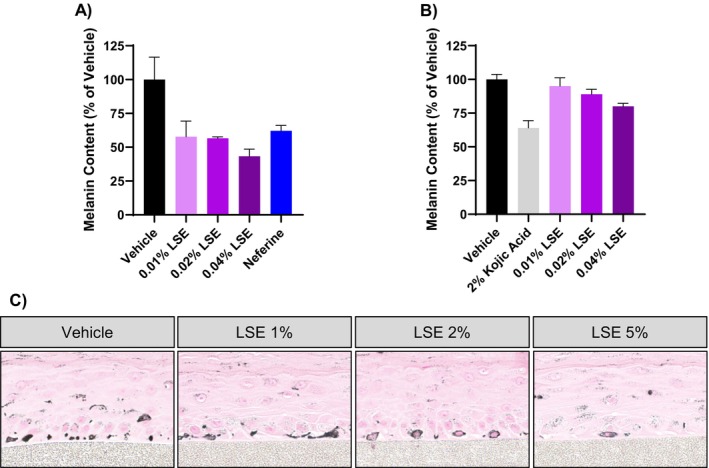
LSE reduces melanin accumulation in HEMn‐DP normal human melanocytes and 3D MelanoDerm cultures. (A) Melanin content of HEMn‐DP cells after treatment as indicated for 7 days. (B) Melanin content of MelanoDerm tissues after treatment as indicated for 15 days. (C) Fontana‐Masson staining of MelanoDerm tissues after treatment as indicated for 15 days.

As MNT‐1 are malignant melanoma cells, we verified that LSE would reduce melanin accumulation in nontransformed cells. LSE treatment reduced melanin accumulation in HEMn‐DP cells, normal human melanocytes derived from a darkly pigmented donor (Figure 5A). In addition, topical LSE treatment in MelanoDerm tissues significantly reduced melanin accumulation after 15 days (Figure 5B). It should be noted that, consistent with previous studies examining compounds effects on melanogenesis, higher concentrations of LSE were used on the melanoderm 3D tissue model than the 2D cell culture systems [23, 24, 25]. These dissimilar concentrations are to account for the differences in permeation and diffusion of the compound between the two systems. Histological assessment with Fontana‐Masson staining also showed a reduction in melanin particles in the basal melanocytes and throughout the tissue (Figure 5C).

**FIGURE 6:**
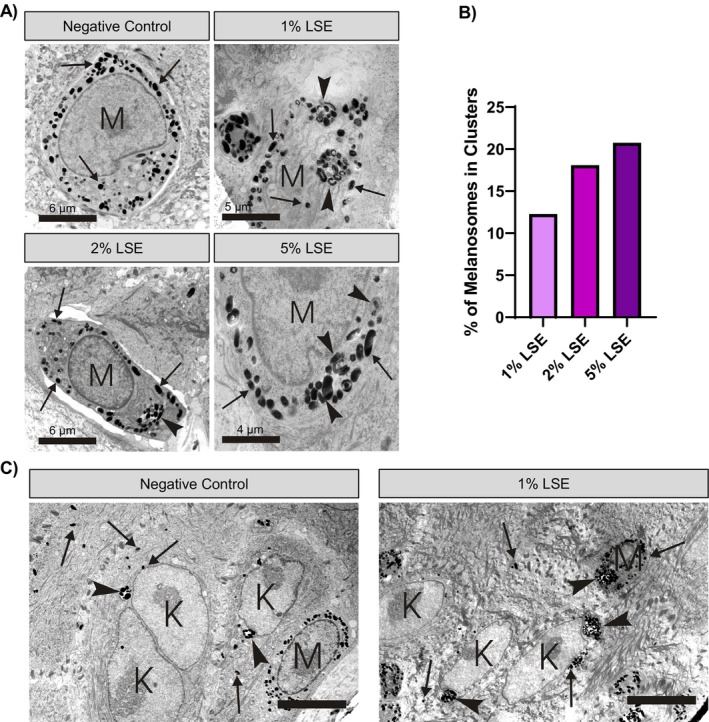
LSE may induce melanosome‐selective autophagy in MelanoDerm cultures. TEM images of basal melanocytes (A), and epidermis (C) consisting of keratinocytes (K) with melanocytes (M) from MelanoDerm cultures treated as indicated for 15 days. Arrows indicate free melanosomes, and arrowheads indicate clustered melanosomes in autophagosomes. (B) Quantification of melanosome distribution showed a dose‐dependent increase in the proportion of melanosomes observered clusered in autophagosomes. Scale bars in (A) represent the distance indicated and scale bars in (C) both represent 12 μm.

To verify the presence of autophagosomes and to gain more insight into how LSE affects melanosome distribution, we performed electron microscopy on LSE‐treated MelanoDerm cultures. In the basal melanocytes of vehicle‐treated tissue, melanosomes were dispersed as individual organelles throughout the cytosol (Figure 6A). In contrast, most (approximately 90%) of the melanocytes treated with LSE showed melanosomes clustered in autophagosomes, visible as membrane‐delimited compartments (Figure 6A). Interestingly, we noted that the autophagosomes induced by LSE contained melanosomes and no other organelles, suggesting that autophagic degradation was selective toward melanosomes. Quantification of melanosome distribution within the melanocytes showed a dose‐dependent increase in the proportion of melanosomes observed clustered in autophagosomes versus free melanosomes after LSE treatment (Figure 6B). Enlarged clusters of melanosomes were also observed in the keratinocytes adjacent to the basal melanocytes (Figure 6C).

**FIGURE 7:**
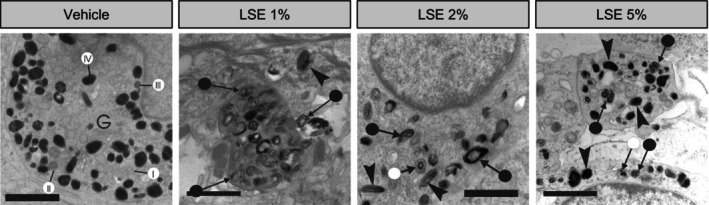
LSE interferes with normal melanosome maturation. TEM images of MelanoDerm cultures treated as indicated for 15 days. Representative melanosomes from each stage are called out in the vehicle‐treated condition (I–IV) around the Golgi apparatus (G). Melanocytes treated with LSE demonstrated a mix of morphologically normal dark melanosomes (arrowheads), plus numerous irregularly shaped melanosomes with partially pigmented matrixes (arrows with closed circles), peripheral spacing between the partially pigmented matrix and the limiting membrane (arrows with open circles). Scale bars represent 3 μm.

Upon closer examination, we observed changes in melanosome morphology with LSE treatment. Melanocytes in the vehicle‐treated MelanoDerm cultures predominantly contained stage IV melanosomes, with occasional stage I, II, and III melanosomes in the vicinity of the Golgi apparatus (Figure 6A). In contrast, melanocytes in MelanoDerm cultures treated with LSE displayed a mix of morphologically normal and aberrantly structured melanosomes. Some melanosomes were irregularly shaped in LSE‐treated melanocytes, as opposed to the uniformly oval or round melanosomes observed in the vehicle‐treated melanocytes (Figure 7). In addition to this, some melanosomes appear more enlarged and darker following 5% LSE treatment than 1% LSE treatment (Figure 7). Other irregularities observed with LSE treatment included partially pigmented melanosomes with distinct melanin foci and melanosomes with peripheral spacing between the matrix and limiting membrane (Figure 7). Altogether, these observations suggest that LSE, in addition to stimulating autophagy, may also partially disrupt melanosome biogenesis and maturation.

## 4 Discussion

In this study, we showed that neferine and liensinine, bisbenzylisoquinoline alkaloids enriched in LSE, reduced melanin accumulation in cultured melanocytes without affecting the expression of melanogenesis‐related genes or tyrosinase activity. Instead, we found that these compounds increased autophagic flux which corresponded to the reduction of pigmentation. We showed that LSE similarly reduced melanin accumulation and activated autophagy in melanocytes as well as MelanoDerm tissue, an in vitro co‐culture model that more closely resembles human skin as compared to 2D monoculture. TEM images revealed that LSE‐induced autophagy was selective for melanosomes, and that the structures of melanosomes in the melanocytes of treated MelanoDerm cultures were frequently aberrant. Together, these results suggest that LSE reduces skin pigmentation by promoting melanin degradation and potentially impeding normal melanosome formation.

With regards to skin pigmentation, a decrease in autophagic activity is associated with hyperpigmentation, and activating autophagy can reduce skin pigmentation by promoting melanosome degradation in melanocytes and keratinocytes [7, 8, 26]. Upon treatment of melanocytes with neferine, we did not see changes in melanogenic gene expression or tyrosinase activity, which is required for melanin formation and occurs in late stage melanosomes. Melanosomes have four maturation stages: stages I and II melanosomes establish their structural foundations, while stages III and IV melanosomes develop melanin and are enriched with pigmentation [6]. These data therefore suggested that increased melanin degradation, rather than decreased production, was the cause. This mechanism is distinct from what was previously proposed for LSE and other lotus extracts, whereby the reduction in melanin has been associated with the suppression of melanogenesis‐related genes and tyrosinase inhibition [11, 13]. Upon autophagy initiation, LC3 is processed into LC3‐I in the cytosol, lipidated to form LC3‐II, and then incorporated into autophagosomal membranes. Once the autophagosomes fuse with the lysosome, the contents, including LC3, are degraded into their constituent macromolecules and recycled for reuse. Thus, the accumulation of LC3‐II represents an increase in the number of autophagosomes present in cells [27]. While the induction of autophagy by neferine and liensinine has been reported, our study shows a previously unknown function for these compounds as depigmenting agents [16, 28].

Although it was already known that LSE contained a considerable amount of neferine and liensinine, the application of LSE as an autophagy inducer in melanocytes had not been previously explored. Moreover, the mechanism by which neferine and liensinine induce autophagy in melanocytes was unclear. Beclin1 is a multi‐domain protein with a large interactome and is a central regulator in autophagy initiation. Beclin1 is activated by ULK1, leading to the production of phosphatidylinositol‐3‐phosphate, a key lipid molecule for autophagosome assembly. Beclin1 is differentially regulated by two important signaling pathways involving mTORC1 and/or AMPK [21]. The specific increase in Ser90 phosphorylation, concomitant with the absence of change in Ser15 phosphorylation, suggests that AMPK, and not mTORC1, contributes to elevated autophagy seen with LSE treatment [22]. A recent study showed that neferine and neferine‐rich extracts from the germ of lotus seed increased autophagic flux in aging fibroblasts, consistent with what we observed in MNT‐1 melanocytes. They also report an increase in beclin1 phosphorylation through a distinct mechanism involving DAPK1 [29]. It is possible that DAPK1 may contribute to autophagy induction in melanocytes; however, further studies are needed to fully elucidate the mechanism of neferine, liensinine, and LSE‐induced autophagy in this context.

Visible skin color is affected by the amount and distribution of melanin in epidermal keratinocytes, and the degradation of melanosomes in melanocytes and keratinocytes by autophagy is one way that apparent pigmentation can be regulated [7, 30]. Because neferine was previously shown to stimulate autophagy in several different cell types, we suspect that LSE would act similarly in keratinocytes. While we did not directly measure autophagy in keratinocytes, we show that the overall melanin content of MelanoDerm cultures, a pigmented skin model that contains both melanocytes and keratinocytes, was decreased from LSE treatment. In addition, we observed large clusters of melanosomes in the keratinocytes of LSE‐treated MelanoDerm cultures by TEM; these large clusters could represent either melanosomes engulfed by autophagosomes or the membrane‐bound clusters of melanosomes typically observed in lighter skin [31].

A notable observation was that autophagosomes induced by LSE treatment in melanocytes appeared to exclusively contain melanosomes, through identification of dark melanosome‐like vesicles which suggested that LSE‐induced autophagy was selective toward melanosomes. In contrast to the bulk autophagocytosis that occurs in response to nutrient deprivation, targeted autophagy uses specific receptors and adapter proteins to promote the recruitment of cargo into autophagosomes. Selective autophagy has been described for some organelles, melanosome‐specific autophagy (melanophagy) has been observed in some studies [32]. Future studies should extend this initial characterization of LSE selectivity using antibodies targeting LC3BII to validate whether LSE treatment results in autophagy strictly for melanosomes. However, very little is known about the underlying mechanisms, and no specific adapters have been discovered that are necessary for the recruitment of melanosomes into autophagosomes. Further understanding of LSE's actions in melanocytes may reveal key regulators involved in melanophagy. Altogether, our results suggest a link between LSE, autophagy, and the regulation of skin pigmentation.

Because LSE contains several bioactive compounds other than neferine and liensinine, it is possible these components may confer complimentary or synergistic activities against pigmentation that are not limited to the induction of autophagy. Other studies have reported that LSE moderately inhibited tyrosinase activity in vitro, and α‐MSH stimulated melanogenesis in murine melanoma cells [12]. While we did not evaluate melanogenic gene expression or tyrosinase activity after LSE treatment in melanocytes, we observed structural aberrations by TEM indicative of impaired melanin production. Under normal conditions, melanosomes exist in four maturation stages: Stage I melanosomes are spherical with wispy unorganized filaments, stage II melanosomes are oval with filaments arranged in a lattice‐like pattern, stage III melanosomes contain melanin deposits on the still visible filament structures, and stage IV melanosomes are fully pigmented and filled with melanin. Specifically, LSE‐treated melanocytes exhibited melanosomes with irregular shape and pigment density. The irregular foci of melanin and the proximity of filament structures to these foci implied that pigment deposition on the melanosome matrix was impeded after treatment with LSE. Taken together, our ultrastructural observations suggest that LSE may also impair melanosome biogenesis and complete pigment synthesis, which could contribute to LSE‐induced depigmentation.

There are limitations regarding these study data that pose some outstanding questions. It is unknown if pigment loss induced by LSE treatment occurs primarily in an autophagy‐dependent manner, which may be further complicated by findings that autophagy regulators are involved with pigment synthesis and melanosome transport, in addition to pigment degradation [20, 33]. Experiments using genetic interference and chemical inhibitors of autophagy are needed to determine the contribution of autophagy to LSE‐induced depigmentation. It remains unclear whether the induction of autophagy is a primary cause or a resulting consequence of LSE treatment. While we showed that neferine did not directly inhibit tyrosinase catalytic activity or melanogenic gene expression, we did not perform the same assays with LSE. Additionally, we used a cell‐free assay performed on purified tyrosinase, which would not capture potential cellular mechanisms affecting overall tyrosinase activity, such as posttranslational processing, delivery to melanosomes, or accelerated proteolysis. Future studies are needed to determine the activities attributed to neferine versus other constituents of LSE and to understand the defects observed in pigment production.

In conclusion, this study shows that neferine, liensinine, and LSE which are rich in neferine and liensinine, reduce pigmentation, possibly due to induction of autophagy and subsequent melanosome degradation. Moreover, our findings suggest that LSE may also impact melanosome biogenesis and maturation. We surmise that using purified compounds of interest to demonstrate mechanism, as we did here for neferine and liensinine, and manufacturing extracts enriched for such compounds may improve efficacy for natural products used in cosmetic applications. Further studies are warranted to explore the molecular mechanisms underlying the activity of LSE and its potential for treating hyperpigmentation.

## Author Contributions

Neil Poloso, Kuniko Kadoya, Prithwiraj Maitra, and Rahul Mehta were involved in study design. Mikhail Geyfman, Robin Chung, and Raymond Boissy were involved in acquisition of data. All authors contributed to the analysis and interpretation of data. All authors have been involved in reviewing and revising the manuscript and have read and approved the final manuscript.

## Conflicts of Interest

This study was sponsored by Allergan Aesthetics, an AbbVie company. All authors meet the ICMJE criteria for authorship, and neither honoraria nor other forms of payment were made for authorship. Financial arrangements of the authors with companies whose products may be related to the present report are as follows, as declared by the authors: R.B. is an investigator and consultant for Allergan Aesthetics, an AbbVie company. M.G., N.P., K.K., P.M., and R.M. are employees of Allergan Aesthetics, an AbbVie company. R.C. and R.M. were employees of Allergan Aesthetics, an AbbVie company during the time of study.

## Supporting information


Table S1.

## Data Availability

The data that support the findings of this study are available from the corresponding author upon reasonable request.
