# Supplementary material for: Lotus Sprout Extract Induces Selective Melanosomal Autophagy and Reduces Pigmentation
Source: J Cosmet Dermatol. 2024 Sep 21;24(1):e16587. doi: 10.1111/jocd.16587 (PMC11743048; doi:10.1111/jocd.16587)
Supplement: Supplementary file 1 — Table S1. [file JOCD-24-e16587-s001.docx]

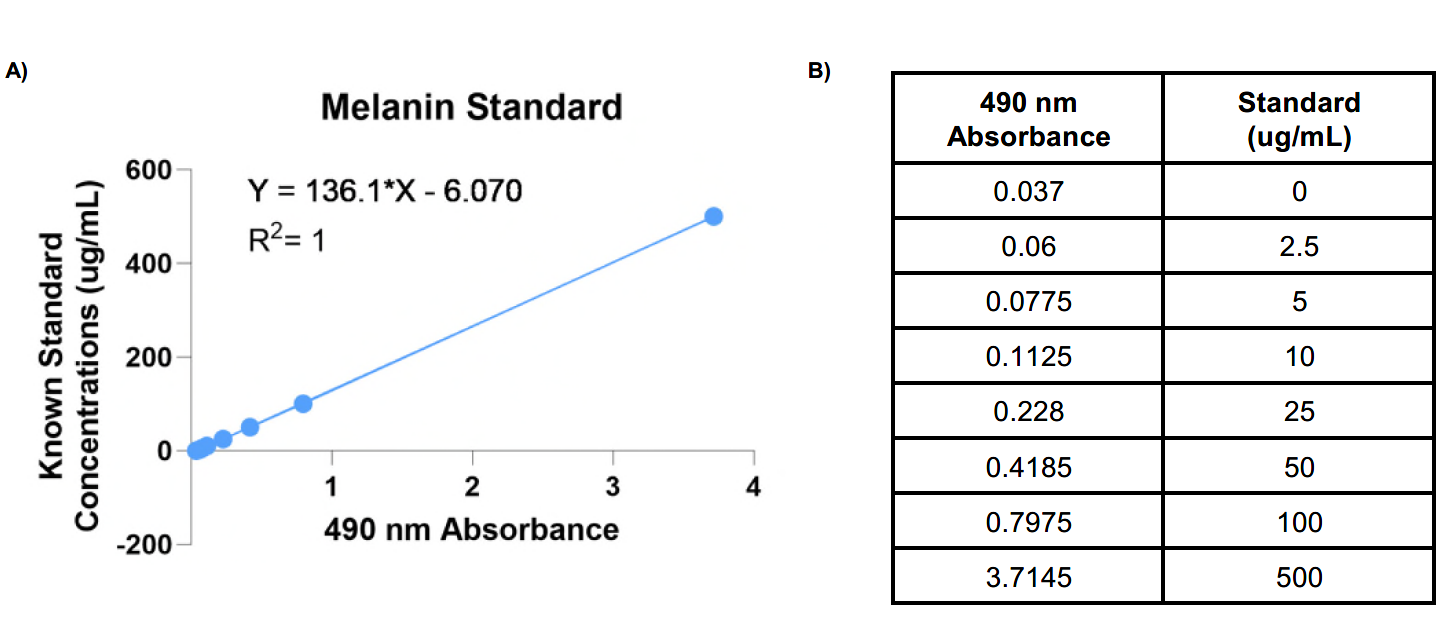


Supporting Information 1. Melanin Standard Curve for Melanin Quantification. Absorbance values for a series of synthetic melanin standards were measured at 490 nm with a Spectramax 340PC microplate reader to assess melanin content in experimental samples. Representative graph (A) and listed values (B) of a standard curve from synthetic melanin (Supp. Info 1).
